# Supplementary material for: Math and language gender stereotypes: Age and gender differences in implicit biases and explicit beliefs
Source: PLoS One. 2020 Sep 8;15(9):e0238230. doi: 10.1371/journal.pone.0238230 (PMC7478909; doi:10.1371/journal.pone.0238230)
Supplement: S1 File — (PDF) [file pone.0238230.s001.pdf]

Supporting Information

Manuscript: Math and language gender stereotypes:
Age and gender differences in implicit biases and explicit beliefs
Authors: Heidi A. Vuletich, Beth Kurtz-Costes, Erin Cooley, & B. Keith Payne

Contents

Supplemental Analyses 1

Implicit Sports Biases. 1

Explicit Gender Beliefs. 3

Correlations between explicit and implicit measures 5

Stimulus Materials 6

Affect Misattribution Procedure (AMP). 6

Explicit Stereotypes 11

Supplemental Analyses

Implicit Sports Biases. An alternative interpretation of our results regarding math and language implicit biases among youth ages 8-15 is that the AMP was measuring generalized gender biases (e.g., girls = good) rather than domain-specific biases (e.g., girls = good at math). To test this possibility, we examined girls' and boys' implicit biases regarding sports ability (one of the domains included in the study, but not the focus of our main report). To assess implicit biases regarding sports ability in boys and girls, we conducted a 2(Participant Gender) x 3(Age Group) x 2(Prime Gender) ANOVA, with Participant Gender and Age Group as between-subject factors, Prime Gender as a within-subjects factor, and implicit scores as the dependent variable. Our results yielded a significant main effect of Prime Gender, $F(1, 255) = 5.44$, $p = .021$, $\eta^2 =$

.02, which was qualified by a significant Participant Gender x Prime Gender interaction, $F(1, 255) = 15.02$, $p < .001$, $\eta^2 = .06$. As displayed in S1 Fig. below, the mean comparisons of the proportion of girls and boys associated with “good at sports” suggests that boys of all ages showed an implicit bias in favor of boys ($M_{(target\ girls – target\ boys)} = -.07$, $p < .001$, 95% *CI*s = [-.10, -.04]), whereas girls of all ages did not show a gender bias ($M_{(target\ girls – target\ boys\ )} = .02$, $p = .282$, 95% *CI*s = [-.02, .05]). These results are in contrast to our findings regarding math and language implicit biases, which show girls as having an implicit in-group bias and boys showing no bias. These divergent results in two different areas of achievement (sports and academics) suggest that the AMP was sensitive to domain category.

In addition, we conducted implicit/explicit correlations between sports implicit biases and explicit beliefs and found a small, but significant positive correlation ($r(260) = .15$, $p = .014$), perhaps due to the less sensitive nature of stereotypes about sports abilities.

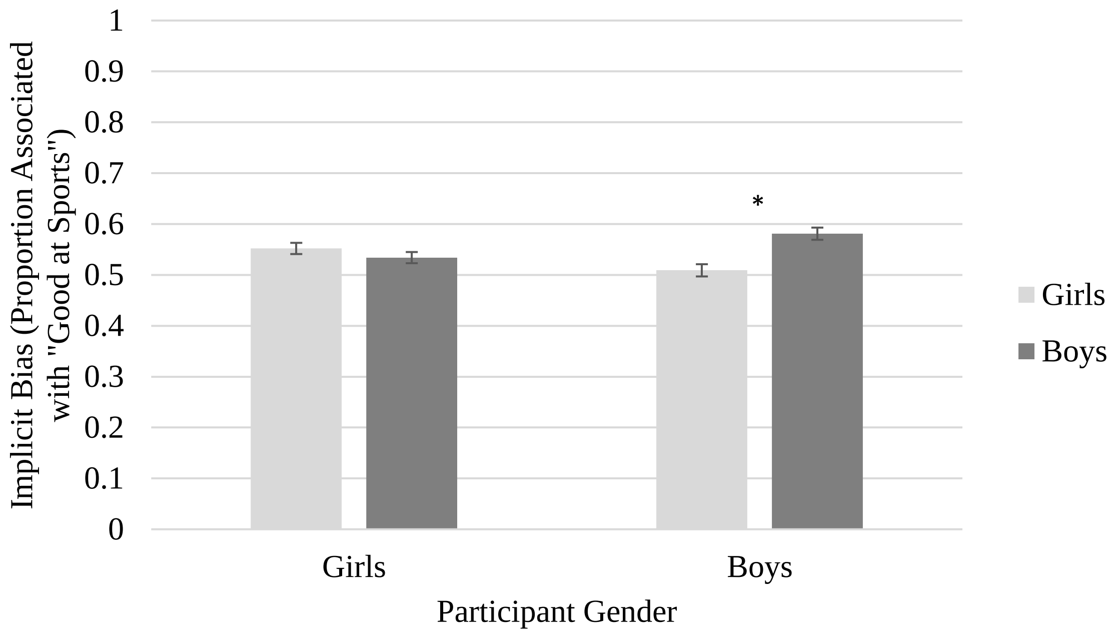

S1 Fig. Estimated Marginal Means for Implicit Bias Scores by Participant Gender. Values indicate the proportion each prime gender associated with “good at sports.” Bars represent standard errors.

Explicit Gender Beliefs. To assess gender differences in children's explicit beliefs regarding math and language ability in boys and girls, we conducted a 2(Participant Gender) x 3(Age Group) x 2(Academic Subject) x 2(Target Gender) ANOVA, with Participant Gender and Age Group as between-subject factors, Academic Subject and Target Gender as within-subject factors, and explicit scores as the dependent variable. Significant main effects are described in the main text. We also found the following significant two-way interactions: Participant Gender x Target Gender [$F(1, 258) = 5.21$, $p = .023$, $\eta^2 = .02$] and Target Gender x Academic Subject [$F(1, 258) = 20.40$, $p < .001$, $\eta^2 = .07$]. These two-way interactions were qualified by three-way interactions, as described in the main text.

Though the four-way interaction that we hypothesized was not significant, perhaps due to low power, we conducted pairwise comparisons with Bonferroni correction to directly test our hypothesis that the youngest children would favor their own group in math (see S1Table). Our hypothesis was partly supported; elementary-school girls reported girls as being better than boys at math ($M_{(target\ girls\ –\ target\ boys)} = 17.51$, $p < .001$, 95% *CI*s = [11.21, 23.80]). Elementary-school boys were neutral regarding gender differences ($M_{(target\ girls\ –\ target\ boys)} = -0.77$, $p = 821$, 95% *CI*s = [-7.46, 5.92]), but they gave boys a significantly higher rating in math than elementary-school girls gave boys ($M_{(girl\ participants\ –\ boy\ participants)} = -15.66$, $p < .001$, 95% *CI*s = [-22.96, -8.36]). Our hypothesis regarding language was that children of all age groups would favor girls over boys in language ability, given cultural stereotypes emphasizing girls' success in language domains combined with gender differences in academic performance. Our hypothesis was partly supported; with the exception of elementary-school-aged boys (who were neutral), children favored girls over boys in language ability. Statistics are presented in S2 Table.

S1 Table. Pairwise Comparisons of Explicit Math Scores by Age Group and Participant Gender.

Participant Gender	Age group	*Mean Diff (Girls - Boys)*	*SE*	*p*	*95% CI*	*n*
Girls	Elementary School	17.51*	3.20	<.001	[11.21, 23.80]	53
	Middle School	1.39	4.66	.766	[-7.78, 10.56]	25
	High School	3.59	2.98	.229	[-2.28, 9.47]	61
Boys	Elementary School	-0.77	3.40	.821	[-7.46, 5.92]	47
	Middle School	7.27*	3.59	.044	[0.20, 14.53]	42
	High School	1.91	3.88	.623	[-5.73, 9.55]	36

Note. *Diff= difference*, *SE* = standard error, *p* = probability value, *CI* = confidence intervals, *n* = number of participants. Adjustment for multiple comparisons: Bonferroni. Mean differences represent how much more competent participants rated girls to be in math compared to boys. Asterisks indicate that girls were rated as more capable than boys.

S2 Table. Pairwise Comparisons of Explicit Language Scores by Age Group and Participant Gender.

Participant Gender	Age group	*Mean Diff* *(Girls - Boys)*	*SE*	*p*	*95% CI*	*n*
Girls	Elementary School	18.34*	2.86	<.001	[12.70, 23.98]	53
	Middle School	14.7*	4.17	.001	[6.16, 22.58]	25
	High School	13.62*	2.67	<.001	[8.37, 18.87]	61
Boys	Elementary School	3.03	3.04	.320	[-2.96, 9.01]	47
	Middle School	9.62*	3.22	.003	[3.29, 15.95]	42
	High School	15.94*	3.47	<.001	[9.11, 22.78]	36

Note. *Diff= difference, SE* = standard error, *p* = probability value, *CI* = confidence intervals, *n* = number of participants. Adjustment for multiple comparisons: Bonferroni. Mean differences represent how much more competent participants rated girls to be in language compared to boys. Asterisks indicate that girls were rated as more capable than boys.

Correlations between explicit and implicit measures. Correlations between pairs of implicit scores and pairs of gender group competence scores, split by participant gender, are presented in S3 Table. Implicit scores for these correlations were the proportion of items in which [girls; boys] were associated with the "good in" prompt; for explicit group competence, we used the average of the two explicit items for each gender. Our results mirror the pattern of correlations when scores are collapsed across gender (analysis reported in the main manuscript). All explicit gender group competence ratings were positively associated. For example, youth who rated boys as highly competent in math also tended

to rate boys as highly competent in language, and youth who rated boys as competent in language tended to also rate girls as capable in language. In contrast, implicit scores were positively correlated within gender, with the exception of a significant positive correlation between “good at” associations of girls in language and boys in math among girl participants.

S3 Table. Correlations among implicit bias scores (above the diagonal) and explicit gender group competence (below the diagonal), split by participant gender.

Participant Gender	Score	Math-Boys	Math-Girls	Language-Boys	Language-Girls
Girls	Math-Boys	—	.016	.281**	.177*
	Math-Girls	.087*	—	-.012	.321***
	Language-Boys	.689***	.300***	—	-.134
	Language-Girls	.308***	.503***	.306***	—
Boys	Math-Boys	—	.073	.255**	.068
	Math-Girls	.266**	—	.086	.209*
	Language-Boys	.628***	.384***	—	.114
	Language-Girls	.335***	.495***	.388***	—

Stimulus Materials

Affect Misattribution Procedure (AMP). Participants saw the instructions shown in the section below prior to each block, which consisted of 40 trials. For each trial, there was a brief presentation (200 ms) of a photo on the computer screen. Following the photograph, a black and white pattern (125 ms) and a Chinese symbol were presented (150 ms). A black and white pattern then appeared until the participant made a response. The sequence of photographs was

randomized within each block, and the sequence of domains (language, math, sports, science[1]) was randomized across participants. The photos were selected based on a pilot study to ensure that photos of the two genders did not differ on perceived attractiveness, age, or mood.

Instructions for language arts. The instructions for math were exactly the same, except that "math" substituted "language arts."

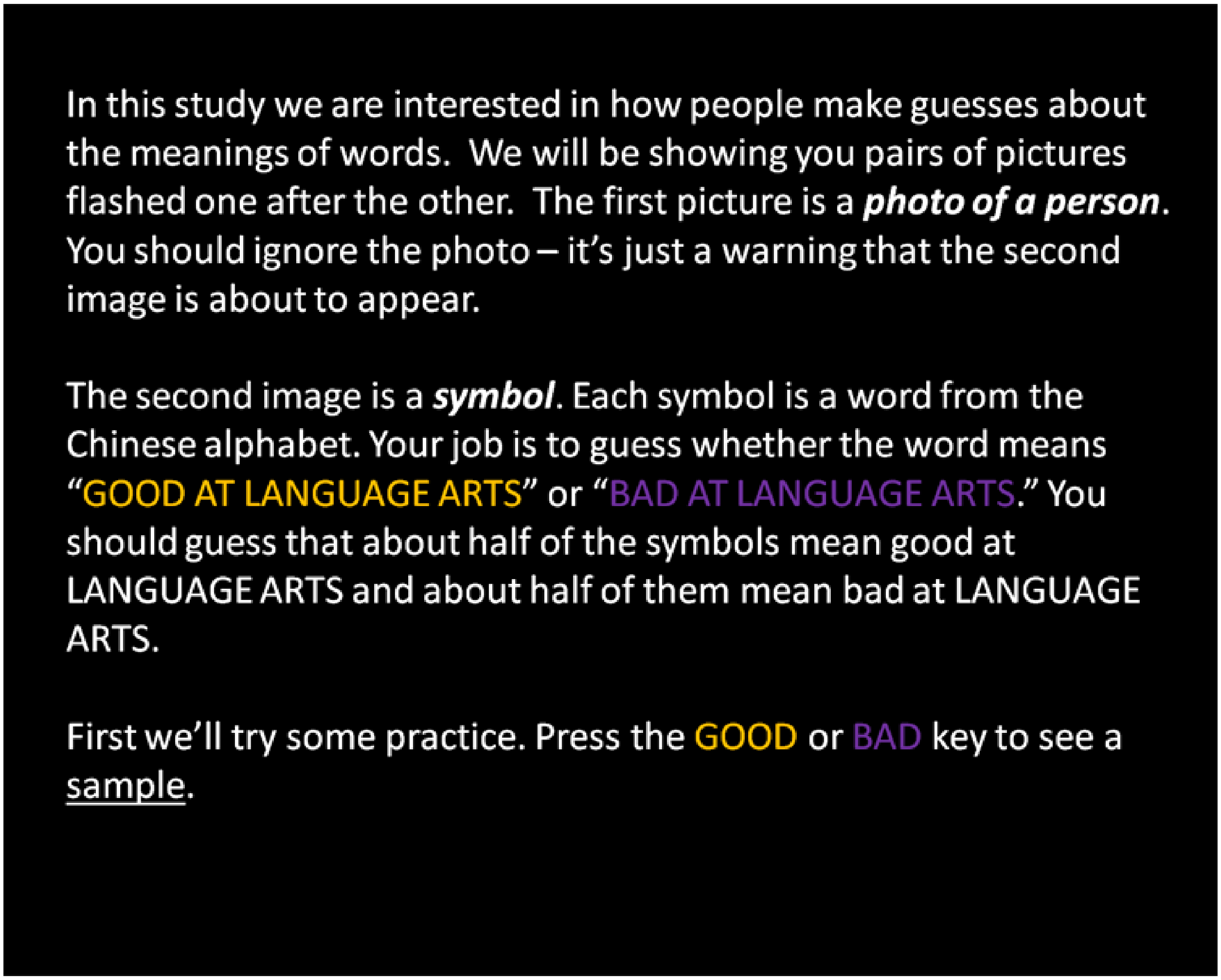

[1] The science block was introduced half-way through data collection and was completed by only 140 out of 270 participants. Of the participants who completed the science block, only 7 were in elementary school, 45 were in middle school and 88 were in high school. The science and sports blocks were not the focus of our main report.

See how fast the pictures flashed? Now you make a guess. You should press the “GOOD” key if you guess the symbol means GOOD AT LANGUAGE ARTS and press the “BAD” key if you think it means bad at language arts.

We do not expect you to know the correct answers; please just use your feelings to make a guess. Remember: you should ignore the photos of the people and just guess the meaning of the Chinese symbol.

Now let’s do a few more practice guesses. Put your fingers on the GOOD and BAD keys and press either one to begin.

Now you're finished with practice. Next you will see more of the same kinds of picture pairs. This part of the task will last about 3 minutes. Now, please find the GOOD and BAD keys and get ready. When you are ready to begin, please press either key.

Black and white pattern.

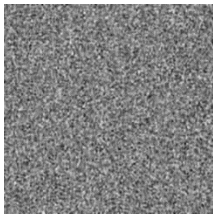

Photographs of boys and girls.

Due to copyright restrictions, these photos are only available upon request and will be shared for personal use only.

Chinese symbols.

勿 午 为 瓦 兔

土 田 它 巳 术

手 氏 矢 尸 身

勺 山 三 入 肉

日 犬 曲 且 齐

朋女牛乃目
木皿民米门
矛吕力来亢\

Explicit Stereotypes. Youth used a visual analog scale (VAS), consisting of a 100 mm horizontal line, to indicate with a vertical mark how well they thought boys or girls performed on a specific academic subject and how difficult they thought boys or girls found the subject. They could place a mark anywhere on the line, which allowed them to give very low or high ratings without having to choose the extreme option, as is the case with Likert scales. This attribute of VAS lines is important when measuring beliefs or attitudes that are sensitive to social desirability effects, such as stereotypes. Other items included in this measure, but not the focus of this report, were group competence regarding sports, science, grades, smartness, making friends, and music. In addition to measuring explicit beliefs about group competence by gender, there were also measures assessing group competence by race. The explicit survey also included other measures not the focus of this report.

VAS Practice Items

For each item below, put a mark (|) to indicate how well you do at each activity. You can make your mark anywhere on each of the lines. The lines in the center of the scales below show you where the middle is. Remember for each one, you can put a mark anywhere on the line you want to show if you're not at all good at that activity, if you're a little bit good, average, good, very good, etc.

1. I think that in **DRAWING** I do this well:

Not well at all ←---------------------------------|---------------------------------→ very well

2. I think that in **COOKING** I do this well:

Not well at all ←---------------------------------|---------------------------------→ very well

Group Competence Measure

For each item below, put a mark (|) to describe **GIRLS**. Remember that you can make a mark anywhere you want on each line.

I think that in **MATH** girls do this well:

Not well at all ←---------------------------------|---------------------------------→ Very well

I think that in **READING** girls do this well:

Not well at all ←---------------------------------|---------------------------------→ Very well

I think that girls find **READING**:

Very hard ←---------------------------------|---------------------------------→ Very easy

I think that girls find **MATH**:

Very hard ←---------------------------------|---------------------------------→ Very easy

For each item below, put a mark (|) somewhere on the line to describe **BOYS**. Remember that you can make a mark anywhere you want on each line.

I think that in **MATH** boys do this well:

Not well at all ←----------------------------------|----------------------------------→ Very well

I think that in **READING** boys do this well:

Not well at all ←----------------------------------|----------------------------------→ Very well

I think that boys find **READING**:

Very hard ←----------------------------------|----------------------------------→ Very easy

I think that boys find **MATH**:

Very hard ←----------------------------------|----------------------------------→ Very easy
