# Supplementary material for: Math and language gender stereotypes: Age and gender differences in implicit biases and explicit beliefs
Source: PLoS One. 2020 Sep 8;15(9):e0238230. doi: 10.1371/journal.pone.0238230 (PMC7478909; doi:10.1371/journal.pone.0238230)
Supplement: Attachment — Submitted filename: Response to reviewers.pdf [file pone.0238230.s002.pdf]

Editor's Comment #1: My main question had to do with this measure (see also Review 1, point 4). I found that this stereotyping measure was quite cleverly designed. It did not initially map onto my vision for this type of AMP. I therefore found myself wondering whether it has previously been validated with adults? If so, this should be made clear in the introduction. If not, I wonder what evidence there is that children could complete this measure successfully and that they believed the cover story. At the risk of seeming self-serving, you might consider referencing previous research that has used the AMP with children to provide some initial evidence (see Perszyk et al., 2019; Williams et al. 2016; Williams & Steele, 2019). However, these papers either validate the AMP or make use of a child-friendly AMP to examine racial bias. This stereotyping measure is different in many ways. For one, I would guess that some children might question whether a language can really have roughly 20 words for good at math, 20 words for bad at math, 20 for good at language arts and 20 for bad at language arts when our own language has no single word to describe these? Were there any questions to assess the believability of this measure and/or did your instructions make it clear what language the symbols were from (perhaps multiple languages?)? I agree with Reviewer 2 that the additional blocks might have influenced the effects and/or might provide additional insights.

Response: This version of the AMP, focusing specifically on academic stereotypes, has not been validated in adults or children, though we did conduct a pilot study to determine the most appropriate presentation time for each stimulus. We have clarified the point about validity in the introduction and also added the suggested references discussing previous use of the AMP with children (bottom of pg. 13). Thank you for this suggestion.

We did not assess the believability of the measure, in part because research with adults has shown that people have difficulty introspecting on how they judged the stimuli and tend to confabulate reasons (Payne et al., 2013). Moreover, the instructions were ambiguous as to whether the symbols actually meant "good at math" or "bad at math" (same for language arts). Our instructions stated that we were interested in how people make guesses about the meaning of words, and that they should guess that about half the symbols meant "good at math" and about half meant "bad at math." We said that they should use their "feelings" to make a guess. We are now more explicit about the wording of these instructions in the Method section (pg. 19).

Regardless of children's interpretation or suspicion of the instructions, the systematic differences that we found between boys' and girls' ratings of different gendered primes were *inconsistent* with their controlled responses as measured by the explicit measure. Yet, these systematic differences suggest that children did not respond completely at random either. One possibility, as raised by the editor and one of the reviewers, is that children ignored the academic subject and simply responded with their gender biases of girls versus boys as "good" or "bad." To test this possibility, we ran additional analyses examining children's sports implicit biases. As with math and language implicit biases, we found a significant gender x prime gender interaction. However, the pattern of results was different compared to the academic biases. For sports, boys showed an implicit bias favoring boys in sports, whereas girls did not show a gendered bias. As a reminder, for math and language, girls had shown an implicit bias favoring girls and boys had not shown a bias favoring either gender. These results suggest that the AMP was measuring domain-specific implicit biases rather than general gender biases. These results are now included in the supplemental information (and briefly mentioned in the main manuscript, bottom of pg. 24).

Editor's Comment #2: In addition, in my own work using the AMP with children, we found that even with extensive instructions and reminders, a portion of our child participants needed to be removed either because of patterned responding (e.g., the same key or alternating keys on each trial), or because when they were questioned at the end of the measure they reported judging the primes and not the neutral stimuli (despite repeated reminders and extensive instructions/explanations that we wanted them to rate the neutral stimuli). I noticed that you had excluded very few participants – were there any checks of this sort? (see also Reviewer 1, minor point 1 regarding exclusions). One concern, of course, is that this is instead some of type of explicit measures, at least for some portion of your child participants. Another concern, particularly given the use of "good" or "bad" is that this a measure of gender bias (see Baron et al. for related findings of gender bias in children using the IAT).

Response: We have now included a statement clarifying that none of our participants met our exclusion criterion, which was pressing the same key on all trials (pg. 20). As for pressing alternating keys on each trial, we are not able to identify this type of patterned responding because the stimuli were randomly ordered for each participant and during each block. The software that we used did not record the order of stimuli presentation for each participant.

Please see our response to comment #1 above regarding the possibility that the AMP, in this context, was a type of explicit measure. First, children's math and language implicit biases were not correlated with their explicit reports. Second, their implicit biases in sports showed a different pattern of bias by gender group, suggesting that the measure was sensitive to domain. Third, the implicit sport biases of children showed a small, but significant, correlation with their explicit beliefs about sports competence (r = .15). These results suggest that this version of the AMP was measuring systematic differences in automatic associations to academic subjects based on gender categories, and that for less socially sensitive stereotypes (like those about sports) there is a small correlation between implicit and explicit measures. We now include these robustness checks in the supplemental information. Thank you for raising this important point and encouraging us to run additional analyses.

We also included a new section in the introduction dedicated to discussing the AMP in detail (pg. 12).

Editor's Comment #3: I also found myself having questions about the analyses. First, why is race treated as a covariate? What happens if it is not included as a covariate? Second, I wondered why both math and arts were not included in the same model. This, of course, would make for a more complex design (a 2x3x2x2 design), but it seems more appropriate. In fact, part of me wondered whether separating one of the other between subjects variables would make more sense than separating out this within-subjects measure in order to simplify the design. For example, looking at each age group separately or each gender group separately. Ideally, of course, this would have all been decided a priori, but looking at your data it would seem that one might draw different conclusions depending on how these data are analyzed.

Response: We are happy to clarify these points. Race was treated as a covariate because academic stereotypes can sometimes differ by race categories. Unfortunately, we did not have adequate numbers of youth in each racial group to test race differences, so we controlled for race instead. The results remain the same when race is removed from the analyses, except that the explicit three-way academic subject x target gender x grade interaction was reduced to a significant academic subject x prime gender interaction where children across all three grades (not just elementary school) favored girls in math. We now leave out race as a covariate.

We initially chose to analyze academic subjects in separate models to simplify the analyses, avoiding the possibility of a four-way interaction. However, we appreciate the editor's point that it is more appropriate to keep this within-subject variable in one model. We now report our results using a 2x3x2x2 design. The findings remain the same.

Editor's Comment #4: One additional concern, that is noted by both reviewers (point 1 for each), is that I am not quite sure what to make the results. If the measures are sound, then these results contribute to what are already mixed findings in childhood regarding the development of a math-gender stereotypes. I would like to see their comments addressed and specific points in the discussion toned down. For example, I do not agree that, based on your data, the results "are consistent with the idea that implicit linkages between girls and language domains were driving those earlier findings."

Response: Thank you for raising this concern. We have now clarified our findings' theoretical contributions. First, we clarify that the vast majority of previous studies examining math and language implicit biases in children or adolescents have used the IAT (pg. 8). These studies show a clear pattern of results among girls of all age-groups: girls show a male-math bias and this bias may predict math-related outcomes such as enrollment intentions for math courses and math self-concepts. However, because of the nature of paired categories in the IAT, these results could be due to girls having a strong association of math with boys, a strong association of girls with language, or both. We found only two studies that used implicit measures that did not confound math and language associations. In one, girls demonstrated an *own*-gender bias across all grades (4-6). In the other, 9th grade girls did not show a bias for either gender. In other words, the math-male bias among girls may only appear when math and language gender associations are confounded. The implication is that girls may not, in fact, have a negative association of their own gender to math. Rather, when math and language are directly contrasted, they might preferentially associate their own gender with language. Our study is important because it adds evidence that girls have an in-group math bias as well as an in-group language bias (summary at the end of pg. 11). Our findings highlight the need for more research that uses implicit measures that do not confound math and language biases to understand if and for what groups these findings replicate.

Editor's Comment #5: Related to my previous point, I felt that the introduction was not as comprehensive as it might have been. There are papers that provide evidence that the math-gender IAT predicts math-related outcomes. These are not consistent with the notion that these biases are driven by a language-girls association. This should be acknowledged. I also found the comment in the discussion that "very few studies have examined both implicit biases and explicit beliefs within the same sample" to be inaccurate, as most do. What they might not do, is report the correlation between the measures.

Response: We have now expanded the introduction to describe previous explicit and implicit findings in more detail, and in particular, findings that link math-gender IAT to math outcomes (bottom of pg. 9). Likewise, in the general discussion, we expand upon why we believe that strong language-girls associations (as compared to math-girls associations), might still predict academic outcomes in math-related domains. In page 33, we added:

Though, at first glance, our results might not appear consistent with gender disparities in STEM careers, they are revealing in that they support recent theoretical frameworks suggesting that girls opt out of math, not due to perceived deficit in math ability compared to boys, but due to perceived strength in

language ability over math ability. For example, a large international study of 15-year-old students found that girls' comparative advantage in reading as opposed to math can largely explain gender disparities in intentions to pursue math-related careers (67). In that study, girls who were found to be good at math were more likely than boys to be even better at reading than at math. The gap between math and reading performance accounted completely for gender differences in math self-concept, interest in math, and attitudes towards math. Other studies have also found that intra-individual contrasts of math and language abilities predict STEM disparities. In a longitudinal study of twelfth grade students, those with high ability in both math and language (more girls than boys) were less likely to pursue STEM careers than those with high ability in math and moderate ability in language (6). Although cultural stereotypes can still be detrimental to girls insofar as they elicit stereotype-threat effects (68) or signal lack of belonging (69), our results imply that girls hold positive associations about their gender group across both math and language ability, consistent with models that depict girls as having more choices in their pursuits, rather than being bound by real or perceived ability constraints. Research that distinguishes between math and language implicit beliefs, then, is important because it can lead to different conclusions about the type of interventions that might be effective for reducing STEM disparities.

We have also deleted our comment about other studies not measuring implicit biases and explicit beliefs within the same sample.

Reviewer 1

Reviewer 1, Comment #1: In the introduction, the argument that social identify theory and ingroup gender preferences drive children's responses fits for published literature on girl participants (i.e., girls rated as better at academics than boys) but not boys (who also seem to rate girls as better at academics than boys). Is this the best theory to use to explain the whole pattern of results? The authors do a more comprehensive job in the discussion at explaining why the pattern of results might reflect ingroup preference for boys as well. Perhaps this should be worked into the intro as well?

Response: We have expanded and restructured much of the introduction to make the pattern of results (or lack thereof) for past studies clearer. We also revised the way we discuss specific theories for explaining children's responses on explicit measures. More specifically, we acknowledge different theoretical perspectives (including social identity theory), but point out that published research on this topic has yielded inconsistent findings (pg.6-7). Our view is that multiple factors likely influence explicit reports due to participants' ability to control their responses. This challenge with explicit measures highlights the advantage of using implicit bias measures.

Reviewer 1, Comment #2: A summary of the overall pattern of results for literature on explicit and implicit stereotypes would be helpful. Overall, I get the sense that the results are not consistent. Do the results of the current study aim to clarify the field in any way?

Response: Thank you for this suggestion. We have included summary statements for the explicit (pg. 7, line 138) and implicit findings (pg. 11, line 238). For explicit findings, there is no overall pattern. We clarify this point and suggest reasons for why this might be true. For implicit findings, the pattern is very consistent for studies using IAT measures; youth (girls in particular) show a stereotypical math-male bias and language-female bias. Studies that did not rely on IAT, but used measures that disambiguate math/language biases, did not find math-male biases among girls. Our study aims to reconcile these existing, conflicting findings by providing evidence that measurement differences could account for the

seemingly inconsistent findings in the implicit bias literature (specific to math/language biases). We find that girls show a counter-stereotypical math-female bias. These findings are important for our understanding of how automatic associations about math and language ability influence motivation and career trajectories.

Reviewer 1, Comment #3: I think the paper would benefit from greater clarity regarding for whom the stereotype applies. Research (e.g., Steele, 2003) suggests that children are more likely to apply math-gender stereotypes to adult (but not child) targets. It seems that the literature reviewed is focused on child targets (perhaps explaining the discrepancies in results from different studies). If cultural stereotypes are more readily applied to adults, this has implications for the hypotheses specified page 10 (line 212) as child targets were included in the AMP, and for conclusions regarding the explicit measure.

Response: Thank you for pointing out this important distinction (i.e., to what age group the stereotype applies). We chose to focus on child targets for two reasons. First, as you pointed out, much of the prior literature examining youths' academic gender stereotypes has used child targets. Second, youths' views of the competence of social group members—in this case, their perceptions of the abilities of boys and girls who are roughly their age—are known to be related to their perceptions of their own abilities, their domain-specific interests, and other motivational variables. We have added this point as a study limitation in the Discussion as excerpted below (pg. 35):

A third limitation of the study is that we assessed students' implicit associations and explicit beliefs regarding gender differences in the abilities of youth targets, but not adults. Some studies have shown that children apply cultural academic stereotypes to adults more readily than they do to children (e.g., 33). Although the explicit reports of adolescents in our sample favored girls in math, youth may have favored men over women had we used adult targets. Such results would reflect gender differences in career choices that favor men in STEM domains and would also be consistent with stereotype threat effects that show performance decrements for women when gender identity is made salient in test situations.

Reviewer 1, Comment #4: Has the AMP been used to measure stereotypes previously? My concern (especially with the younger children) is that the valance of the response categories trumped the stereotype component. Can this be disentangled?

Response: This is an excellent point. We now include supplemental analyses showing that boys favor boys in sports, rather than girls, whereas girls show no bias. These results suggest that the AMP was testing domain-specific gender associations. Please see our response to Editor's Comment #1.

Reviewer 1, Comment #5: The implicit and explicit results have a similar pattern in that girls are deemed better in language than boys. Why is this said to reflect "cultural knowledge" for the explicit results but "academic success" for the implicit results? Is it possible that implicit and explicit measures reflect the same underlying constructs, but differences in measurement variability prevent strong correlations from emerging?

On a related point, Page 9, line 186. "Children, however, may not yet have learned the cultural stereotypes and so may vary in awareness". Or it could be that children have learned the stereotype,

but have not yet internalized it to the point it can be automatically activated by attitude object. What are the implications of these possibilities for the hypotheses and results?

Response: We agree with the reviewer that our results do not speak to the specific constructs underlying the observed biases. We changed the language in our discussion to reflect their speculative nature. We also added a sentence in the Discussion regarding the possibility that measurement might have led to the lack of correlation between implicit and explicit stereotypes as excerpted below:

It is also possible that the lack of correspondence in our study might be linked to our choice of measures, and that a different methodological approach might yield significant relations between children's implicit associations and their explicit reports (p. 34, line 674).

Reviewer 1, Comment #6: 2.2% of sample of Asian heritage. Did these participants have any familiarity with Chinese symbols. If so, should they be removed?

Response: Only six participants reported their race as Asian-American. If we exclude these participants, the results remain exactly the same; therefore, we retained their observations.

Reviewer 1, Comment #7: What were the correlations for the four prime-gender scores? (page 20)

Response: We now report these correlations in Table 7.

Reviewer 1, Comment #8: Tone down language around conclusions. For example, p 21 line 328; p 23, line 345. Other studies (using the IAT) have demonstrated age-related differences in implicit biases.

Response: We have made these suggested changes.

Reviewer 2

Reviewer 2, Comment #1: It would help if the authors could make greater sense of the implicit data from boys. That is, these findings seem to contradict past published work where boys show an implicit gender stereotype. Is there a way to compare the strength of the egalitarian associations with math and language to see if the effect is stronger in one direction? There are now a number of papers by Cvencek, as well as those who have done stereotype threat work (Tomasetto, Steele etc) arguing that in some way shape or form boys have a gender stereotype in this domain. Is there something unique about how the IAT measures bias that might make it a more suitable measure in this case? Of course, the data are what they are but I think much more attention should be given to this contradictory finding both in terms of possible methodological explanations as well conceptual. Related, can the authors report more info on average latencies with the AMP? It might help to understand how implicit these responses likely were.

Response: We now clarify that several past studies have also failed to find stereotypic implicit biases in boys. In fact, the findings among boys are much more inconsistent than those among girls. That said, we have also incorporated the references suggested above which document gender stereotypes among boys and have worked to clarify that we are unsure about why boys and girls show dissociative implicit biases (pg. 12):

We were agnostic about the implicit biases of boys, as previous findings have been inconsistent and do not clearly favor one theoretical account over another. The dissociative processes by which girls and boys form automatic associations is in itself interesting, but not the subject of this report.

We still maintain the following:

*The view of academic success as a feminine trait may have led girls in the present study to show implicit biases favoring girls in both domains, whereas for boys, those views may have been tempered by a tendency to show in-group preference, resulting in their egalitarian scores on the task. (*bottom of pg. 32)

As for the associations being stronger in the direction of math or language, we can now speak to that question. We changed our analyses such that math and language are included in the same model (a 2x3x2x2 design). The three-way interaction (i.e., academic subject x prime gender x gender) that might reveal differences in the relative strength of math versus language associations among boys was not significant. Finally, we cannot report information on average latencies because the AMP does not measure reaction times. Rather, it measures the proportion of targets that the participants rate as “good at” or “bad at” math/language when preceded by a photo of either a boy or a girl.

Reviewer 2, Comment #2: I didn't follow the arguments the authors made about how the data on the implicit/explicit measures directly speaks to the sources of these stereotypes. That is, to say that if implicit bias is more influenced by cultural messages about stereotypes then they should increase with age doesn't make clear sense to me. And, by contrast, classroom cue sensitivity would lead to no age differences (lines 210-214). First, it's odd that there wasn't a direct measure of sensitivity to cultural stereotypes or some quantification of classroom cues. It was assumed that patterns of bias uniquely are constrained by these cues when there are a multitude of factors that also uniquely shape bias (e.g., surely they interact). Further, it is not clear that being influenced by cultural messages about stereotypes should mean that the bias increases with age (there isn't strong evidence that implicit bias

reflects a cumulative learning model whereby the magnitude of the bias increases with frequency of exposure), there could be sensitive periods for learning biases, etc. Similarly, what's the evidence the present classroom made available diagnostic cues to performance/ability?

Response: We agree with the reviewer that our results do not speak to the specific sources underlying the observed biases. We changed the language in our introduction and discussion to reflect the fact that our data cannot speak to mechanisms. We are also now explicit in the introduction about the fact that multiple factors can influence beliefs and biases. Thank you for raising this point.

Reviewer 2, Comment #3: The authors noted that other school domains were studied but not the focus of the present manuscript. Rarely do I think these additional study data are informative but for the present manuscript I especially think it's informative because it speaks to broader arguments the paper seems to be very focused on - own group bias, internalization/awareness of cultural stereotypes and classroom cues. Do any of the other data not reported shed light on children's more general sensitivities here?

Response: Thank you for this suggestion. We now include supplemental analyses showing that boys and girls have different implicit biases in sports compared to math and language, suggesting that our implicit measure was sensitive to both gender and academic domain. Please see our response to Editor's Comment #1.

Reviewer 2, Comment #4: I think it would be helpful if the authors could include more discussion on the growing stereotype threat literature in this domain as it seems to be particularly informative for our thinking and predictions about the development of gender differences in these academic stereotypes. And, in some cases, may even present contradictory findings that require some explanation.

Response: This is an excellent point. Because stereotype threat refers to performance decrements in a test situation when membership in a negatively-stereotyped group is activated, results of the current study are not directly related to stereotype threat. Nonetheless, we added that point in the Discussion as excerpted below:

Although cultural stereotypes can still be detrimental to girls insofar as they elicit stereotype-threat effects (68) or signal lack of belonging (69), our results imply that girls hold positive associations about their gender group across both math and language ability, consistent with models that depict girls as having more choices in their pursuits, rather than being bound by real or perceived ability constraints (pg. 33, line 255).

Reviewer 2, Comment #5: The authors setup two primary views about measuring bias - importance and limitations for studying both explicit and then implicit bias. This made sense. I got lost a bit when indirect measures were then discussed because, conceptually, I didn't understand where the authors saw indirect measures fitting in the literature - is it a level of analysis like implicit/explicit or is it kinda orthogonal to the implicit/explicit distinction and more of a way to measure things explicitly while reducing some the potential demand characteristics that can plague explicit measures?

Response: Thank you for raising this point. We agree that results from indirect measures are not relevant to the current study and have removed reference to these studies.

Reviewer 2, Comment #6: Lastly, can the authors highlight/note the analyses they were likely underpowered for given then power analysis they did as the effect sizes reported in a number of cases seemed to be below the threshold they set for the study.

Response: We have noted in the Results section the effect that fell below the threshold calculated from our sensitivity analysis. Excerpted from pg 21:

The Age Group x Academic Subject x Target Gender interaction was also significant, $F(2, 258) = 3.51$, $p = .031$, $\eta 2 = .03$. Children favored girls in language across all three age groups. In contrast, in the case of math, only children in elementary school showed a bias, favoring girls over boys in math. Youth in middle school and high school did not show a gender bias in explicit reports of math ability. These results should be interpreted with caution, though, as the effect size is below the threshold calculated by our sensitivity analysis. The four-way interaction was not significant, perhaps due to low power.
