# Supplementary material for: Math and language gender stereotypes: Age and gender differences in implicit biases and explicit beliefs
Source: PLoS One. 2020 Sep 8;15(9):e0238230. doi: 10.1371/journal.pone.0238230 (PMC7478909; doi:10.1371/journal.pone.0238230)
Supplement: Attachment — Submitted filename: Response to reviewers_7.10.20.docx [file pone.0238230.s003.docx]

# Editor’s Comments

**Editor’s comment 1:** I believe that there is tremendous benefit to using a range of measures to gain a deeper understanding of the early development of implicit academic stereotypes. I also appreciate the additional information that you have provided in the supporting information document, which strengthens your interpretation of the data.

**Response:** Thank you for this affirmation of our work. We are glad the inclusion of the additional supporting information was helpful.

**Editor’s comment 2:** I would encourage you to work to address each of the reviewer’s comments, with a focus on additional limitations that will need to be noted in the discussion section. In particular, Reviewer 1 raised two main points that will need to be adequately addressed in the discussion. That is, given the different nature of this particular measure, you cannot conclude that the implicit language-stereotyping effect for girls is driving the math-gender stereotyping on the IAT (more on that below).

**Response:** We agree that this is an important point to address in the discussion. Here’s how we responded to Reviewer 1 regarding that comment:

“We agree that there is no conclusive evidence that implicit language associations were driving the IAT math-gender stereotype findings. Our intention is to highlight the fact that isolating math implicit biases as the mechanism driving IAT results is unwarranted. At a minimum, our study suggests that gender math associations do not override the positive associations that girls have about girls’ general academic performance compared to boys.’ A more generous interpretation of our results suggests that girls’ associate girls (not boys) with good math performance. Either interpretation challenges the assumption that girls’ gender associations to math ability favor boys instead of girls. We have tried to clarify our argument as follows (lines 601-618):

‘An important contribution of the current study is that we used a measure of implicit gender biases in which math and language abilities were not confounded. Most prior research in this area has used the Implicit Association Task, in which the two pairings (e.g., boys-math; girls-language) are not measured independently. Of note, authors of those studies have often interpreted their results as indicating an implicit bias favoring boys in math, and have used their measures to predict math-related outcomes. However, our results suggest that girls hold implicit biases favoring girls in both math and language. In supplemental analyses, we examined whether these associations might have been an artifact of the measure’s sensitivity, which may not have been granular enough to test domain-specific associations, but only broader associations (e.g., “girls = good at academics/school” rather than “girls = good at math). We examined implicit biases regarding sports ability and found a different pattern of results from academic biases. Girls were neutral whereas boys favored boys in sports ability. Although these biases may still reflect a general association between girls and good academic performance, they suggest the AMP was sensitive to domain-category and challenge prior assumptions that girls implicitly favor boys over girls in math. At a minimum, our study suggests that gender math associations do not override the positive associations that girls have about girls’ general academic performance compared to boys.’ Another interpretation is that girls’ associate girls (not boys) with good math performance, in line with their explicit reports.’”

**Editor’s comment 3:** Reviewer 2 raises a number of important points, many of which should at the very least be addressed in the discussion. In particular, the relative lack of exclusion criteria should be discussed relative to other papers that make use of implicit measures with child participants, with a focus on what might have been done to ensure that your effects are not simply the result of a great deal of noisy participants in the data (more on that from me below as well).

**Response:** We apologize for an oversight on our part, which was that the order of responses was recorded by the software used for the implicit task. This information, along with response latencies/reaction times, was in the raw data files (approximately 900 individual files, one for each participant and each subject- math, language arts, sports, and science). These variables were excluded during the original merging process as they did not represent key outcome data. Making use of these disaggregated raw files, we reviewed one block for each participant, looking for a pattern of alternating key presses on all trials. None of the files contained this pattern. Some files contained patches of alternating responses, but none were exclusively alternating. We now note this exclusion criterion in the manuscript and note that none of the participants met it.

**Editor’s comment 4:** The inclusion of the implicit sports biases measure was a great addition. But this led me to wonder what other measures were included in this study. I would request that in the current climate of replicability, these be outlined in the supporting information. Is it possible that some effects did not emerge because children were fatigued by the time they were completing the key measures? Could these effects have been influenced by other measures that they completed prior to the main measures of interest?

**Response:** There was only one other block in the implicit task (for a total of 4), so we have added this information to the main text in line 418: “Two other school domains (sports and science) were included in separate blocks, but are not the focus of this report.” The science block was introduced half-way through data collection and was completed by only 140 out of 270 participants. Of the participants who completed the science block, only 7 were in elementary school, 45 were in middle school and 88 were in high school. We have included this information in the supporting information. Given the short duration of the implicit task, which took about 5 minutes to complete, fatigue is an unlikely factor influencing results. We failed to mention in our initial submission that blocks were counterbalanced across participants, eliminating the possibility of carryover effects. We have added that information in line 419: “Block presentation was counterbalanced across participants.”

**Editor’s comment 5:** I was unclear what the M*diff* score was for the implicit sports bias in the supporting information.  More information on that measure is needed. I would also recommend that this get integrated into the main manuscript as I believe that the measure has the potential to speak against a very clear alternative explanation. However, please see Reviewer 1 as the limitation to this possibility needs to be fully addressed in the discussion.

**Response:** We have now changed the M*_diff_* label to a more clear M_(_*_target girls – target boys_*_)_ label. The score signifies the mean difference between proportion of girl photos associated with “good at sports” and proportion of boy photos associated with “good at sports.” We used the following language to clarify:

“As displayed in S1 Fig. below, the mean comparisons of the proportion of girls and boys associated with “good at sports” suggests that boys of all ages showed an implicit bias in favor of boys (*M _(target girls – target boys)_* = -.07, *p* < .001, 95% *CI*s = [-.10, -.04]), whereas girls of all ages did not show a gender bias (*M _(target girls – target boys )_* = .02, *p* = .282, 95% *CI*s = [-.02, .05]).

After careful consideration, we decided not to integrate the sports measure into our main manuscript. Doing so would require changing the theoretical focus of the paper, shifting attention away from our main contrast of interest—math versus language using a different implicit measure from previous studies—to a focus on the particulars of the AMP in this specific experimental context. A background on sports biases and the developmental changes in those biases is beyond the scope of this paper. Keeping our analyses as they are also makes the comparison to previous studies—all of which have only focused on math and language—more straightforward. Instead, we have dedicated more space to this particular finding in the discussion, drawing the reader’s attention to the supplemental information (lines 607-618):

“In supplemental analyses, we examined whether these associations might have been an artifact of the measure’s sensitivity, which may not have been granular enough to test domain-specific associations, but only broader associations (e.g., “girls = good” rather than “girls = good at math). We examined implicit biases regarding sports ability and found a different pattern of results from academic biases. Girls were neutral whereas boys favored boys in sports ability. Although these biases may still reflect a general association between girls and good academic performance, they suggest the AMP was sensitive to domain-category and challenge prior assumptions that girls implicitly favor boys over girls in math. At a minimum, our study suggests that gender math associations do not override the positive associations that girls have about girls’ general academic performance compared to boys.’ Another interpretation is that girls’ associate girls (not boys) with good math performance, in line with their explicit reports.”

**Editor’s comment 6**: Another way that the positivity bias could be addressed is by examining responses to your different stimuli. I wonder, if you separate the Black and White targets, do you see a race bias? That is, were participants more likely to select “good at” when presented with a White target prime as opposed to a Black target prime? Is this particularly true for the Black male primes and the non-Black participants? (see Perszyk et al., 2019). This might be worth exploring and might provide additional insight that could be included and considered in the supplement.

**Response:** We gave this point a lot of thought. Although running these analyses is possible, we hesitated and ultimately decided not to do so because we do not believe it would help to address the question about a positivity bias. One issue is that academic stereotypes exist in regard to both race and gender. A widespread stereotype in the U.S. is that Black children are worse at academics than White children. Thus, observing a bias in favor of Whites versus Blacks for the academic subjects does not clarify whether the bias is about the general positivity associated with Whites or the specific association that Whites are better than Blacks at math and language. This confound means that the analyses would not yield any additional information to clarify the positivity bias in question.

Although stereotypes regarding sports ability are not confounded with race stereotypes in adults (in general, stereotypes favor Whites over Blacks, but favor Blacks over Whites in athletic ability), it is less clear whether children of all ages demonstrate a positivity bias towards Whites, and even less clear if they show a positivity bias towards Blacks in sports. One study (Williams & Steele, 2019) found that children 9 to 12-years-old did not show an implicit bias in favor of Whites on the AMP. Thus, it is unclear to us how we would interpret any findings from analysis of race bias in academics or sports. Others might find these analyses helpful, however, so we have included a link to our data with this submission and plan to make it publicly available pending publication acceptance: https://osf.io/fv5h8/?view_only=a5e988b47f694da5ad56072fc48a9bc8.

To acknowledge this limitation of our current design, we also note in the Method that although we ruled out a broad positivity bias in favor of girls (girls=good) with the analysis of the sports AMP, we cannot rule out a less general, but still not subject-specific, bias (girls=good at academics) [lines 487-499]:

“An alternative interpretation of our results is that the AMP was measuring generalized gender biases (e.g., girls = good at academics) rather than domain-specific biases (e.g., girls = good at math). To test this possibility, we examined girls’ and boys’ implicit biases regarding sports ability (one of the domains included in the study, but not the focus of this report). We report the results in our Supplementary Information. In short, boys showed evidence of implicit bias favoring boys for the sports domain, but girls showed no implicit bias. Although these results might still reflect generalized biases (i.e., girls = good at academics / boys = good at athletics), they suggest that the AMP was sensitive to domain category and not just valance. In theory, if the instrument distinguishes between broad categories (e.g., sports versus academics), then it can distinguish between the categories specified in the actual task (sports v. math v. language), unless the effects of the specific academic subjects are so small as to be overwhelmed by the broader category. This latter point is in itself informative and contrary to the current understanding of girls’ implicit biases.

**Editor’s comment 7:** The paragraph that starts on line 276 is meant to address my concern that many of your participants might have rated the primes instead of the neutral stimuli. However, I did not find this to really address the concern.  As noted by Reviewer 2 and by me above, the possibility exists that the lack of stereotyping is due to a small effect combined with noise in your child data due to a relative lack of exclusion criteria. Please see the other AMP papers with children (Williams et al., Williams & Steele, Perszyk et al.) for the criteria that were used. If you can apply these, wonderful – if not, this limitation needs to be more fully addressed in the discussion. I will also mention that I found the exclusion criteria description on lines 447-448 to be awkward and would recommend rewording.

**Response:** We have more carefully assessed our raw data for response order and patterns, and we found that none of the participants engaged in patterned responding whereby they pressed alternating keys on all trials (more details about this process in Response to Editor’s comment #3). We have also revised the wording of the exclusion criteria as follows (lines 460-461):

“Our exclusion criteria for the implicit bias task (pressing the same key on all trials or alternating keys on all trials) did not apply to any participant.”

**Editor’s comment 8:** I am conflicted about the analyses that are presented and would suggest that you give some additional thought to them.  In the absence of the four-way interaction, you ultimately do not directly test your hypotheses that, for example, “the youngest age group…(will) favor their own gender in math”, etc. I know that one school of thought is that these direct comparisons should not be made in the absence of the interaction effects. On the other hand, given the complexity of your design and your specific hypotheses, I wondered whether including more direct tests would be helpful in making full sense of these data. I also wondered whether the conclusions that are drawn on lines 584-585 completely align with the analyses that you conduct. I do think that providing access to the data – which I don’t think were submitted, but you note the intention to link to these – might solve this issue. But I would be open to you including some additional analyses in the supplemental materials or main text.

**Response:** Thank you for this excellent point. We have now included more direct tests of our hypothesis in the Supplemental Information and summarized these findings in the main text (lines 536-544). The text added to the Supplemental Information is excerpted below:

“Though the four-way interaction that we hypothesized was not significant, perhaps due to low power, we conducted pairwise comparisons with Bonferroni correction to directly test our hypothesis that the youngest children would favor their own group in math. Our hypothesis was partly supported; elementary-school girls reported girls as being better than boys at math (*M _(target girls – target boys)_* = 17.51, *p* < .001, 95% *CI*s = [11.21, 23.80]). Elementary-school boys were neutral regarding gender differences (*M _(target girls – target boys)_* = -0.77, *p* = 821, 95% *CI*s = [-7.46, 5.92]), but they gave boys a significantly higher rating in math than elementary-school girls gave boys (*M _(girl participants – boy participants)_* = -15.66, *p* < .001, 95% *CI*s = [-22.96, -8.36]). Our hypothesis regarding language was that children of all age groups would favor girls over boys in language ability, given cultural stereotypes emphasizing girls’ success in language domains combined with gender differences in academic performance. Our hypothesis was partly supported; with the exception of elementary-school-aged boys (who were neutral), children favored girls over boys in language ability. Statistics are presented in S1 Table.”

**Editor’s comment 9:** I found ‘road map’ statements to be unnecessary and would recommend that sentences like the one on line 260 be removed.

**Response:** We have deleted these statements.

**Editor’s comment 10:** The reliability of this measure is easily calculated and could be included with the measure. Please include it and remove the justification (on page 14) for not including this.

**Response:** We have made these recommended changes.

**Editor’s comment 11:** What is the theoretical justification for the age grouping that was used?  Please provide some justification.

**Response:** We chose to aggregate scores within the three age groups rather than use age as a continuous variable because the latter approach would have complicated our analyses considerably. The decision to use those specific ages (i.e., 8-10 years, 11-13 years, 14-15 years) was shaped in part by pragmatics, with a concern that each group be large enough to have adequate statistical power, and mostly by developmental considerations. Members of the youngest group (8 to 10 years old) were still in elementary school, where curricula are heavily geared toward mastery/knowledge acquisition rather than performance, children tend to be less aware of individual differences in achievement than at later ages, and in-group bias predominates in judgements of group differences in ability. In middle school (the 11-13 year old group), more curricular tracking occurs, particularly in math classes, and public displays of honors rolls as well as more extracurricular opportunities (athletics; science fairs) as well as normative increases in children’s perspective-taking lead to an increased awareness of group differences in academic choices and skills. As high school students, the 14-15-year-olds were even more aware than middle schoolers of individual and group differences in academic achievement and choices. During high school, some adolescents enroll in many Advanced Placement and honors classes, putting themselves on a trajectory for college admission. Other students take regular track courses. Group differences such as greater representation of boys than girls in AP physics and calculus are highly salient.

**Editor’s comment 12:** I would report on the implicit measure first in the measures and results and then would report on the explicit measure for consistency.

**Response:** We have made the suggested change.

**Editor’s comment 13:** Line 456 – Prime Gender should be Target Gender.  Additional interactions could be fully reported in the supplement.

**Response:** We have made these changes.

**Editor’s comment 14:** Table 7 – it would be interesting to see this for boys and girls separately as well.

**Response:** We now include a table disaggregated by gender in the Supporting Information. The patterns are generally the same.

**Editor’s comment 15:** Overall, I think that there are some real strengths to this manuscript, and I believe that it has the potential to make an important contribution to the field.  I hope that you will decide to address each of these concerns and resubmit the paper for additional consideration.

**Response:** Thank you for considering our manuscript for publication. We hope the changes we made adequately address your concerns and those of the anonymous reviewers.

# Reviewers’ Comments

**Reviewer #1, comment 1**: I believe that this revised manuscript is stronger than the original submission. The comments I raised in my review have been adequately addressed to the extent that the data allows for this. There continues to be many strengths to this paper (i.e., Social ID theory, sample, methods, robust analyses, etc). Most importantly, I agree with the authors that the field would benefit from research that uses diverse implicit measures. The current paper meets that objective without doubt. However, I continue to have two conceptual concerns that may prevent this paper from being publishable in PLOS One.

**Response:** Thank you for this positive assessment and for helping us make this a stronger manuscript.

**Reviewer #1, comment 2:** The authors argue that implicit language associations could be driving the IAT math-gender stereotype findings. I agree. My issue is that there is no data to support this claim (pages 32-33, line 612-618). Instead what we see is a more general implicit girls = good pattern of results. I note that there could still be a contribution to make here in that social id theory can be used to explain this pattern of results, but I am uncertain whether this is novel enough for publication in PLOS One.

**Response:** We agree that there is no conclusive evidence that implicit language associations were driving the IAT math-gender stereotype findings. Our intention is to highlight the fact that isolating math implicit biases as the mechanism driving IAT results is unwarranted. At a minimum, our study suggests that gender math associations do not override the positive associations that girls have about girls’ general academic performance compared to boys.’ A more generous interpretation of our results suggests that girls’ associate girls (not boys) with good math performance. Either interpretation challenges the assumption that girls’ gender associations to math ability favor boys instead of girls. We have tried to clarify our argument as follows (lines 601-618):

“An important contribution of the current study is that we used a measure of implicit gender biases in which math and language abilities were not confounded. Most prior research in this area has used the Implicit Association Task, in which the two pairings (e.g., boys-math; girls-language) are not measured independently. Of note, authors of those studies have often interpreted their results as indicating an implicit bias favoring boys in math, and have used their measures to predict math-related outcomes. However, our results suggest that girls hold implicit biases favoring girls in both math and language. In supplemental analyses, we examined whether these associations might have been an artifact of the measure’s sensitivity, which may not have been granular enough to test domain-specific associations, but only broader associations (e.g., “girls = good at academics/school” rather than “girls = good at math). We examined implicit biases regarding sports ability and found a different pattern of results from academic biases. Girls were neutral whereas boys favored boys in sports ability. Although these biases may still reflect a general association between girls and good academic performance, they suggest the AMP was sensitive to domain-category and challenge prior assumptions that girls implicitly favor boys over girls in math. At a minimum, our study suggests that gender math associations do not override the positive associations that girls have about girls’ general academic performance compared to boys.’ Another interpretation is that girls’ associate girls (not boys) with good math performance, in line with their explicit reports.”

**Reviewer #1, comment 3:** This girls = good issue was raised in the original reviews. To address this the authors examined responses on a Sport-AMP and found that for male participants boys were more positively associated with sport, girls demonstrated no bias (mirroring the academic-related AMPs where boys showed no bias). In my opinion, this analysis does not adequately address the issue. What we may be seeing here is a broader "girls = good at school / boys = good at sports" stereotype that would be consistent with input via cultural exposure. Again, social identity theory could be used for a framework to interpret this pattern of results.
**Response:** We agree with the reviewer that we might be picking up on a more general “girls are good at school, whereas boys are good at sports” stereotype. Indeed, a considerable body of literature (e.g. Heyder & Kessels, 2013; Kessels & Steinmayr, 2013; Wang et al., 2013) has shown strong support for the hypothesis that adolescents view girls as more capable and better suited to achieve academically than boys. However, at the same time, substantial domain-specificity is evident in gender comparisons of youths’ perceptions of their own abilities as well as their perceptions of the abilities of gender groups. Although girls as a gender group are frequently viewed as more capable than boys in school achievement in general, differences tend to be larger in verbal domains, and on average, girls consistently rate themselves as more talented in verbal domains than in math, while boys show the opposite pattern (e.g., Evans et al., 2011; Huang, 2013; Nagy et al., 2010). For this reason, it was important for us to examine responses within academic domains. Please see our response to Reviewer #1, comment 2 for how we addressed this concern in the discussion.

**Reviewer #2, comment 1**: I commend the effort to address so many of the reviewer comments in a thoughtful, clear and concise way. There are some issues that I do think are still quite important to tend to as it bears directly on the framing and claims.

**Response:** Thank you!

**Reviewer #2, comment 2:** The primary focus is to advance our understanding of implicit and explicit gender stereotypes. As such, we want to have some reasonable comfort that the measures are indeed capturing something implicit (and explicit). How can one tell if it's implicit? As we know, there are a variety of different ways to address this, some better than others. But I'm not sure what can be said here as this procedure hasn't been established with children. Not a direct line into what's implicit, an earlier reviewer comment re latency data would be quite useful - particularly if latencies were quite slow. I understand from the response letter that AMPs calculate proportions of response types. Is it the case that the software used really doesn't capture latency data for each trial? I understand the AMP analysis doesn’t incorporate these data but my question is asking whether the software itself has such data. What software was used? Most programs I know capture these data. Assuming this isn’t available, what then can we point to as evidence that this procedure with children has been shown to capture implicit (as opposed to explicit) bias?

**Response:** We appreciate this comment. Because of the popularity of the Implicit Association Test (IAT; Greenwald et al., 1998), which *does* rely on response times to assess implicit biases, reviewers who have reviewed our previous work have often are curious about how we can be sure that the Affect Misattribution Procedure (AMP; Payne et al., 2005) captures implicit attitudes, despite not analyzing response times.

From our perspective, one of the benefits of using an Affect Misattribution Procedure (vs. an IAT) as an implicit measure, is exactly its independence from response times. In particular, response time data often has high variability due to the inevitability of occasional long response times (e.g., a distracted moment) or very quick response times (e.g., pressing buttons as quickly as possible to make it through the tedious task)—both of which can create skewed, noisy data. As a result, analyses on IAT data require data cleaning that involves omitting trials for which response times are too quick or too slow (Greenwald et al., 1998). This noise and variability associated with analyzing response time data would be a key limitation of using an IAT with children.

In contrast, the Affect Misattribution Procedure is a priming measure that does not rely on response times. Instead, the AMP assesses implicit attitudes by pitting intention against a prime's influence. In particular, across trials, participants are told that they need to do their best to ignore the influence of the photo primes (in this case, black and white children), and that these photos are merely a distraction. Instead, participants are told to evaluate the Chinese symbols that follow the primes. Thus, to the degree that people, and in this case children, evaluate the Chinese symbols in systematically different ways based on the photo primes that they were told explicitly to ignore, we can infer that an unintended/implicit attitude has emerged.

That said, the question of whether the AMP assesses implicit (vs. explicit) attitudes was first raised nearly a decade ago (Bar-Anan & Nosek, 2012). Since then, a variety of data have been provided to suggest that the AMP assesses something different than explicit attitudes (see Payne, Brown-Iannuzzi Burkley, Arbuckle, Cooley, Cameron, & Lundberg, 2013). For example, in one study, participants were randomly assigned either to complete the normal version of the AMP (i.e., instructed to ignore the photo primes, but evaluate the symbols); or the reverse (i.e., instructed to intentionally rate the photo primes). Results revealed that responses varied significantly by condition (Payne et al., 2013). This result suggests that, under normal conditions (i.e., when people are instructed to ignore the photo primes and evaluate the symbols), participants are not intentionally rating the photo primes. It follows that any systematic influence of the photo primes on the ratings of the Chinese symbols reflects an unintended/automatic, and thus implicit, attitude.

Finally, there is also a variety of published work that has used variations of the AMP with children (Perszyk, Lei, Bodenhausen, Richeson, & Waxman, 2019; Williams & Steele, 2019). Moreover, children in our studies displayed distinct patterns of responding on the AMP versus our explicit measures. Together these data support the validity of using the AMP to measure implicit associations among children.

We will also note that as the reviewer surmised, the software we used—DirectRT—does record response times by default. However, because we did not intend to analyze response times, and do not have any hypotheses regarding response times, we do not have a sense of how we would analyze response time data in a way that would strengthen the claims made in the paper—especially because there is no precedent for doing so.  However, to address this comment, we have worked to include some of the above reasoning in the paper—both in the introduction when we first speak of the AMP, as well as in the general discussion section of the manuscript—as excerpted below (lines 278-299):

“One potential concern about the AMP is that participants could ignore instructions and directly rate the primes, making the measure more akin to an explicit measure. To address this concern, one study tested two different AMP conditions assessing race implicit bias, one where adult participants were instructed to directly rate the primes and another one where they were instructed to ignore the primes and rate the target stimuli [51]. These two conditions yielded divergent results. The traditional AMP predicted racial bias in an impression formation task, whereas the “explicit” AMP did not, presumably because individuals were motivated to control expressions of prejudice in the explicit condition. Indeed, motivation to control prejudice was related to the explicit AMP but not the traditional one. These results suggest that, under normal conditions (i.e., when people are instructed to ignore the photo primes and evaluate the symbols), participants are not intentionally rating the photo primes. Though these findings were based on adult samples, they likely extend to children, as children tend to have less self-regulatory skills than adults to control their responses [52]. Another study addressing this concern found that adults are not able to accurately introspect on what influenced their response pattern [51], rendering self-reports of this information unreliable. Taken together, these findings suggest that any systematic influence of the photo primes on the ratings of the ambiguous stimuli reflects an unintended/automatic, and thus implicit, bias.

The potential weaknesses of the AMP have been tempered by empirical evidence, and its strengths have been documented extensively. Another strength of the AMP is its reliability. Meta-analytic procedures have shown the AMP to be more reliable than most reaction-time-based measures [48]. There is also evidence that it is a reliable measure for use with children, with good predictive validity [53].”

**Reviewer #2, comment 3:** How come the presentation stimuli times differed from the one AMP study with children to date?
**Response:** We based our presentation times on a pilot study and selected durations that optimized the child’s comfort with the task, but we should note that AMP findings have been shown to be robust across a wide range of stimuli durations both among adults (75 to 1500 ms for primes and 100 to 2000 ms for targets; Payne et al., 2005) and among children (75 to 425 ms for primes and ms 225 to 675 ms for targets; Williams, Steele, & Lipman, 2016). Our presentation times, 150 – 200 ms, fall within the range of presentation times shown to be acceptable with children.

**Reviewer #2, comment 4:** I remain concerned about not checking for whether participants were familiar with the Chinese characters. Imagine, for example, this were taking place today with the rising amounts of overt racism toward China. I could imagine familiarity with the characters could present two issues. 1. Prime negative affect itself. 2. Lead participants to doubt that the characters actually stand for words meaning good/bad at x,y,z if they have a mutual exclusivity hypothesis about Chine language (one character per concept, similar to word learning bias in English and other Western languages). Perhaps this believability doesn't matter?

**Response:** These are important points. First, all the children were aware that the symbols where Chinese. Therefore, any general valanced affect primed by the association to China would have been evenly distributed across participants and across domains. We now include this important point in the Method (lines 414-416): “Participants were told that each symbol was ‘a word from the Chinese alphabet,’ and that we were interested in how people make guesses about the meaning of words.”

The second point speaks to a comment also raised by the editor. We have included the following statements in the Method (lines 420-427): “We do not have information about whether any participants were familiar with the Chinese symbols in our study or on their thoughts about the cover story. However, we should note that the believability of the cover story is superfluous to the task’s objective, which was to rate ambiguous stimuli preceded by a prime. The mechanism by which the AMP functions (i.e., misattribution of affect/semantic content from prime to target) does not depend on construal of the task. Familiarity with the Chinese symbols does present a problem, which is that the symbols would no longer be neutral, weakening priming effects. Thus, though unlikely given our sample’s demographics, we should note that the effects reported here might be underestimates.”

**Reviewer #2, comment 5:** As well, I’m still confused by how we can reasonably conclude this is not an attitude measure toward the primed stimuli (vs stereotypes). Is the study powered for the Sports AMP that was used to demonstrate there isn’t just a positivity bias? Is there a correlation between the two AMPS (presumably there would not be if it were not measuring a general gender good/bad bias)? Was there an order effect with the different AMPs conducted? Is there a domain difference for boys/girls (on ave or by gender) to further help us to see if they’re indeed capturing different constructs?
**Response:** Please see our response to Reviewer 1, comment #2, regarding our edits to address the question of a positivity bias. In short, we have data to suggest that there wasn’t a global positivity bias (girls = good), but we now acknowledge that we can’t rule out a less broad (but still not subject-specific) positivity bias (e.g., girls = good at academics). Because the analysis of sports implicit bias had fewer groups and an equal number of participants compared our main analyses, we can detect effect sizes smaller than the ones reported in the sensitivity analysis in the main text. We are not certain which “two AMPS” the reviewer is referencing, but we’ll note that the proportion of girl photos associated with “good at sports” was positively correlated with the proportion of girl photos associated with “good at” math and language AND positively correlated with the proportion of boy photos associated with “good” at math and language. Finally, as we mentioned in response to an earlier comment, the block order was counterbalanced. We now note this detail in the main text.

**Reviewer #2, comment 6:** I’m puzzled by the very lax exclusion criteria for the AMP. With IAT, for example, exclusion criteria is around 20% errors or greater. Alternating key presses would be missed and this is not uncommon for children. Before I can really make sense of these data I’d want to see much clearer reporting of proportion of trials with one key press, what the range is, SD, etc for age groups. What software was used to run this program?
**Response:** We have re-examined the data for alternating key presses and found no such patterns in the data. None of the participants pressed the same key on all trials. We are now clearer about these exclusion criteria in the main text (lines 460-461): “Our exclusion criteria for the implicit bias task (pressing the same key on all trials or alternating keys on all trials) did not apply to any participant.”

**Reviewer #2, comment 7:** Do we think the mixed results for explicit gender stereotypes reported in the intro is conceptual or methodological? That is, does it reflect variability due to differences in personal and or cultural stereotypes represented by the child or due to methodological differences employed or something else? This would be helpful to discuss perhaps somewhere (but doesn't haven't to be solved here).

**Response:** Because self-reports on socially sensitive topics, like stereotypes, can be subject to self-presentation concerns, it is difficult to discern whether the mixed results are conceptual or methodological. This is precisely the problem implicit measures attempt to circumvent. We have clarified this point in the introduction as follows:

“A problematic aspect of research on academic stereotypes focused on explicit beliefs is that it relies on self-report measures, which allow participants to control their responses. Children may report beliefs that match perceived social expectations or self-presentation goals, regardless of their own beliefs. For instance, 6- to 8-year-old children who thought they were being videotaped suppressed their explicit in-group preference and outgroup prejudice, whereas those who thought the camera was off did not [39]. The mixed findings in explicit attitudes, then, might reflect variability in children’s personal beliefs, but they could also be an artifact of children’s reactance to being questioned about a socially sensitive topic.”

**Reviewer #2, comment 8:** The authors note “We expected girls would show traditional math-male biases if they have assimilated cultural stereotypes that favor boys in math. In contrast, girls would favor girls in math if pervasive differences in academic performance are the primary factor shaping automatic associations about gender and math ability.” Is it possible they could hold both stereotypes and randomly (or non-randomly as with some kind of prime) exhibit one of these stereotypes in the moment? Some children were tested in a local library. Were these more female than male? Were stereotype assessments different here (potentially because of its linkage to language- reading). Given sensitivity on explicit measures to social desirability, was there an effect of experimenter gender on the explicit measure for older children?

**Response:** We are happy to clarify these questions. Speaking to the first point, chronic and situational accessibility of stereotypes is entirely possible, though we wouldn’t necessarily expect systematic differences of either type of accessibility by gender. Regarding the second point, although some children were recruited from local libraries and others were recruited from schools, all children were tested at a library (either school library or public library). Third, we did not track data on the gender of the experimenter, but our procedures were to face away from the participant as they were completing the questionnaire. If the participant had trouble reading, the experimenter assisted by reading the questions using a different survey, thus respecting the participant’s privacy (lines 387-389): “If needed, a researcher assisted younger participants in reading the instructions and questions, but to prevent social desirability effects, the researcher read from a different survey, facing away from the child in order not to look at his or her responses.”

**Reviewer #2, comment 9:** I was to reiterate how important I think it is to measure stereotypes about math/reading/language separately as this confound is really apparent when thinking about the existing findings in this domain with the IAT.

**Response:** Thank you!
